# Supplementary material for: Enhancement of Photoluminescence Quantum Yield and Stability in CsPbBr3 Perovskite Quantum Dots by Trivalent Doping
Source: Nanomaterials (Basel). 2020 Apr 9;10(4):710. doi: 10.3390/nano10040710 (PMC7221998; doi:10.3390/nano10040710)
Supplement: Supplementary file 1 [file nanomaterials-10-00710-s001.pdf]

Supplementary Materials

Enhancement of Photoluminescence Quantum Yield and Stability in $CsPbBr_3$ Perovskite Quantum Dots by Trivalent Doping

Sujeong Jung [1,†], Jae Ho Kim [1,†], Jin Woo Choi [1], Jae-Wook Kang [2], Sung-Ho Jin [3,*], Youngho Kang [4,5,*] and Myungkwan Song [1,*]

[1] Surface Technology Division, Korea Institute of Materials Science (KIMS), 797 Changwondae-ro, Sungsan-Gu, Gyeongsangnam-do, Changwon 51508, Korea; sujeong2@kiost.ac.kr (S.J.); jho83@kims.re.kr (J.H.K.); jinwoo.choi@kims.re.kr (J.W.C.)

[2] Department of Flexible and Printable Electronics, Polymer Materials Fusion Research Center, Chonbuk National University, Jeonju 54896, Korea; jwkang@jbnu.ac.kr

[3] Department of Chemistry Education Graduate Department of Chemical Materials Institute for Plastic Information and Energy Materials, Pusan National University, Busan 46241, Korea

[4] Materials Data Center, Korea Institute of Materials Science (KIMS), 797 Changwondae-ro, Sungsan-Gu, Gyeongsangnam-do, Changwon 51508, Korea

[5] Department of Materials Science and Engineering, Incheon National University, Incheon 22012, Korea

* Correspondence: shjin@pusan.ac.kr (S.-H.J.); youngho84@inu.ac.kr (Y.K.); smk1017@kims.re.kr (M.S.); Tel.: +82-(0)51-510-2727 (S.-H.J.); +82-(0)32-835-8606 (Y.K.); +82-(0)55-280-3686 (M.S.)

† These authors contributed equally to this project.

Received: 17 March 2020; Accepted: 4 April 2020; Published: date

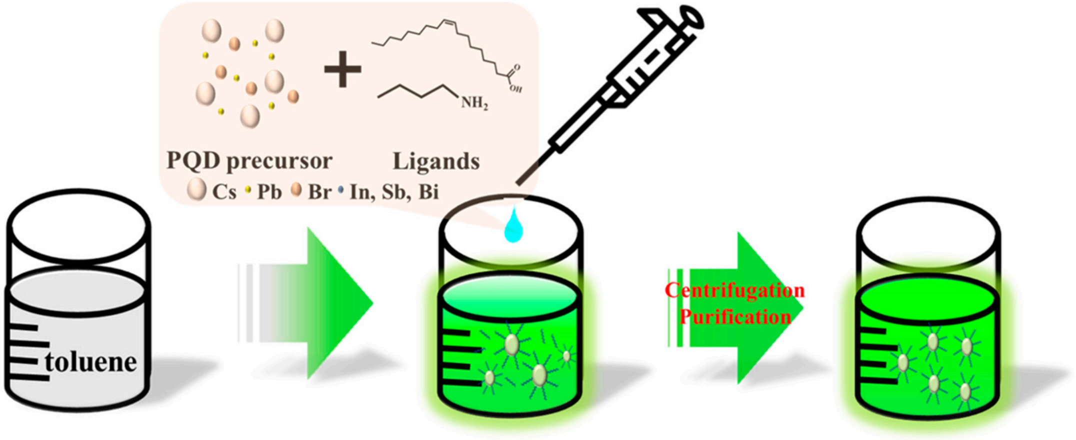

Scheme S1. Schematic of $CsPbBr_3$ synthesis *via* LARP method.

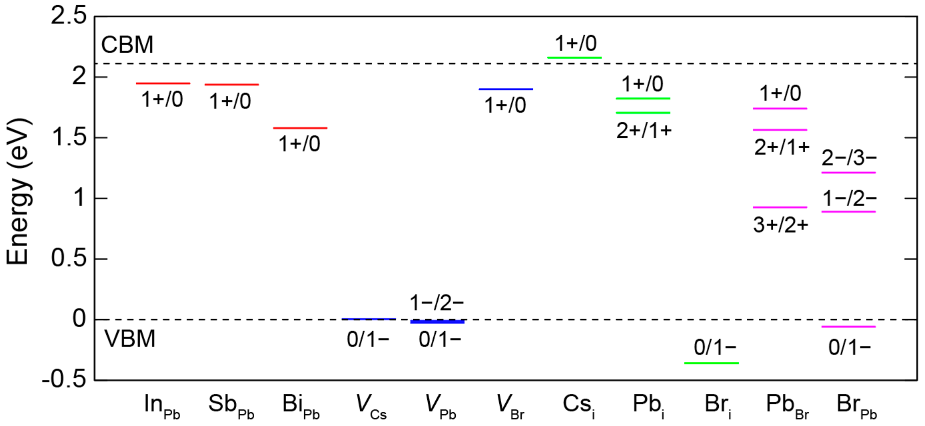

Figure S1. The position of the thermodynamic defect level of ε((1 +)/0) for In_{Pb}, Sb_{Pb}, and Bi_{Pb} as well as native defects such as vacancies and interstitials.

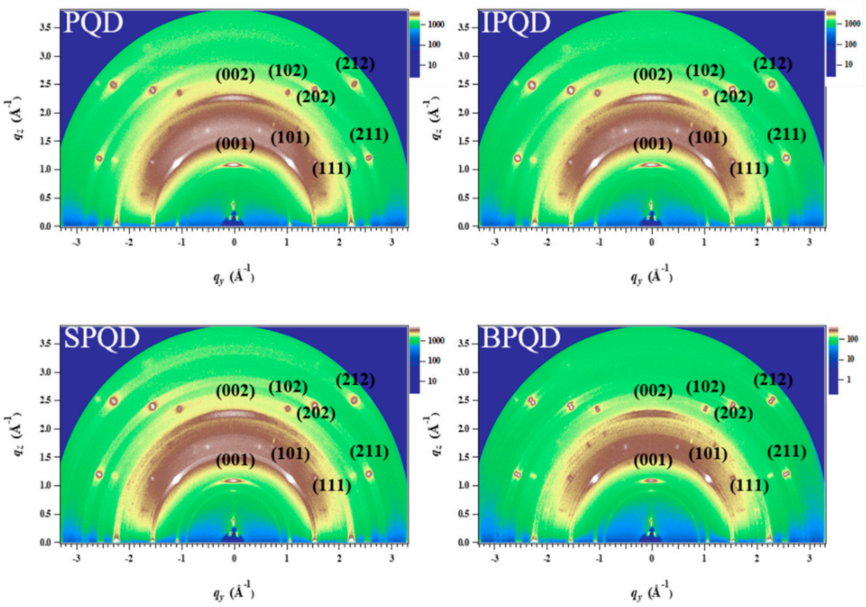

Figure S2. GIWAXS images of pure and 3 mol% In, 3 mol% Sb, and 1 mol% Bi substituted $CsPbBr_3$ perovskite QDs.

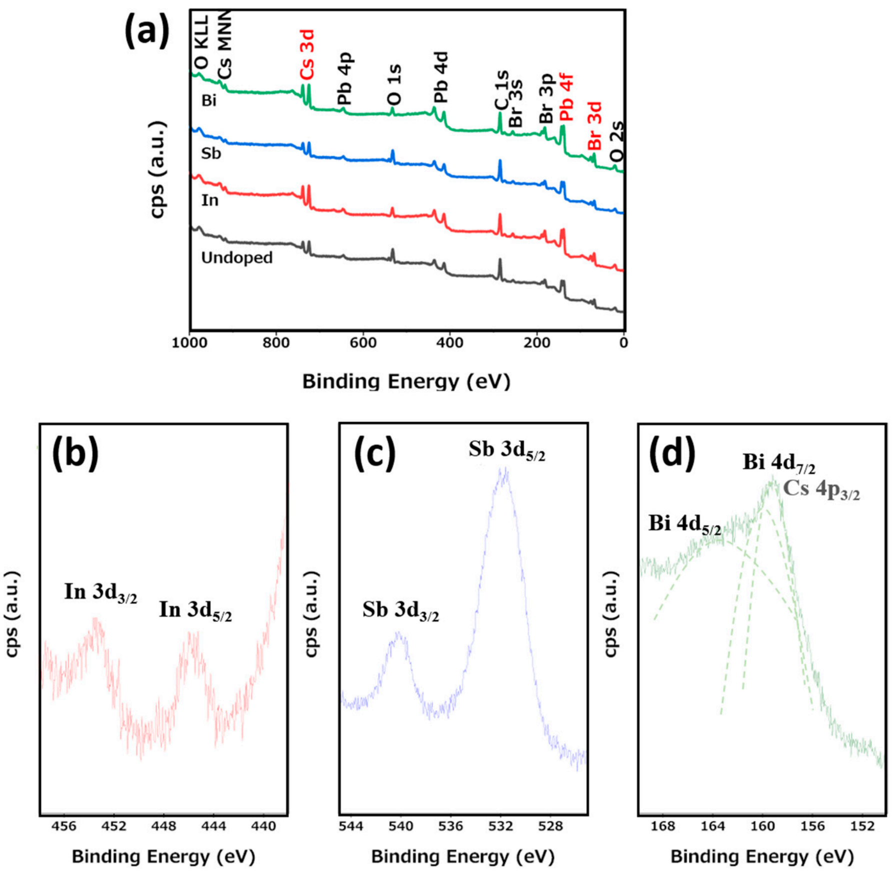

Figure S3. XPS spectra of (a) the pure and doped QDs consist of Cs, Pb, Br, C, and O, (b) In-, (c) Sb-, (d) Bi- substituted $CsPbBr_3$ QDs.

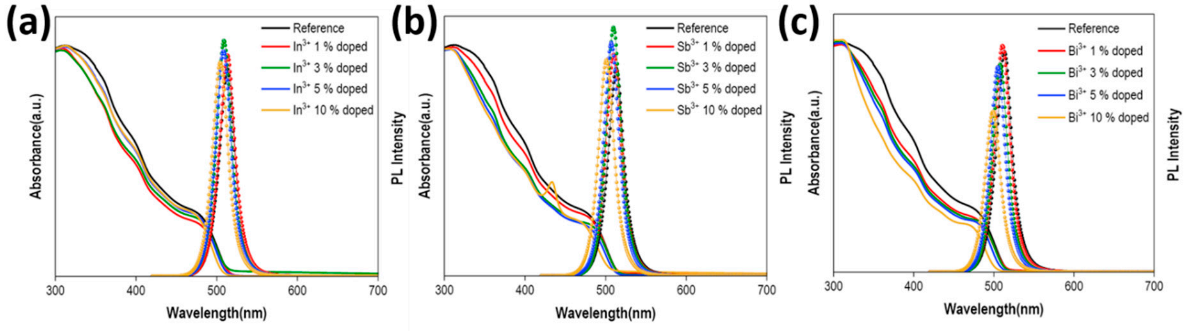

Figure S4. UV-Visible absorbance and PL emission spectra of (a) In- (b) Sb- (c) Bi- substituted $CsPbBr_3$ perovskite QDs with various doping concentration.

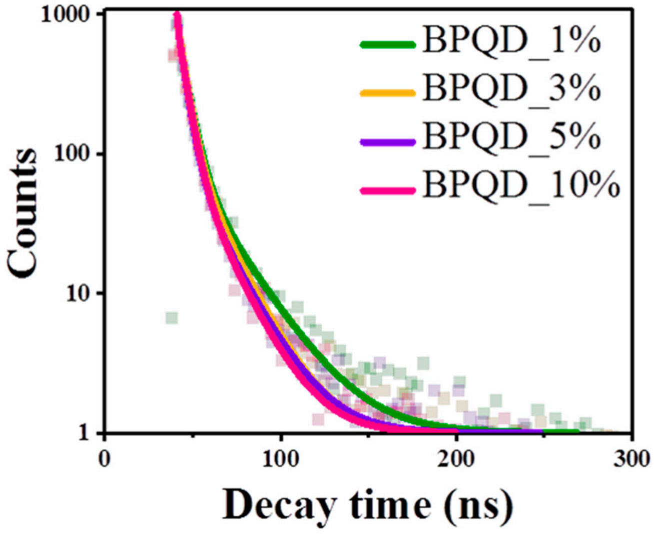

Figure S5. Time-resolved PL decay with fitting curve of 1 mol% Bi ion substituted $CsPbBr_3$ perovskite QDs.

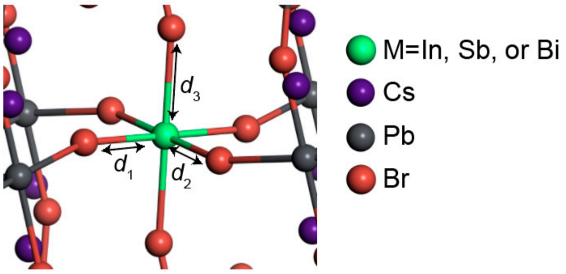

	In		Sb		Bi		Pb
	0	1+	0	1+	0	1+	
d_1 (Å)	2.877	2.679	2.797	2.771	2.97	2.844	2.985
d_2 (Å)	2.879	2.682	2.796	2.773	2.988	2.844	2.982
d_3 (Å)	2.991	2.711	3.166	2.765	2.987	2.835	2.979

Figure S6. Schematic of the atomic structure and the information of lattice parameters of $CsPbBr_3$ perovskite QDs with substituted defects.

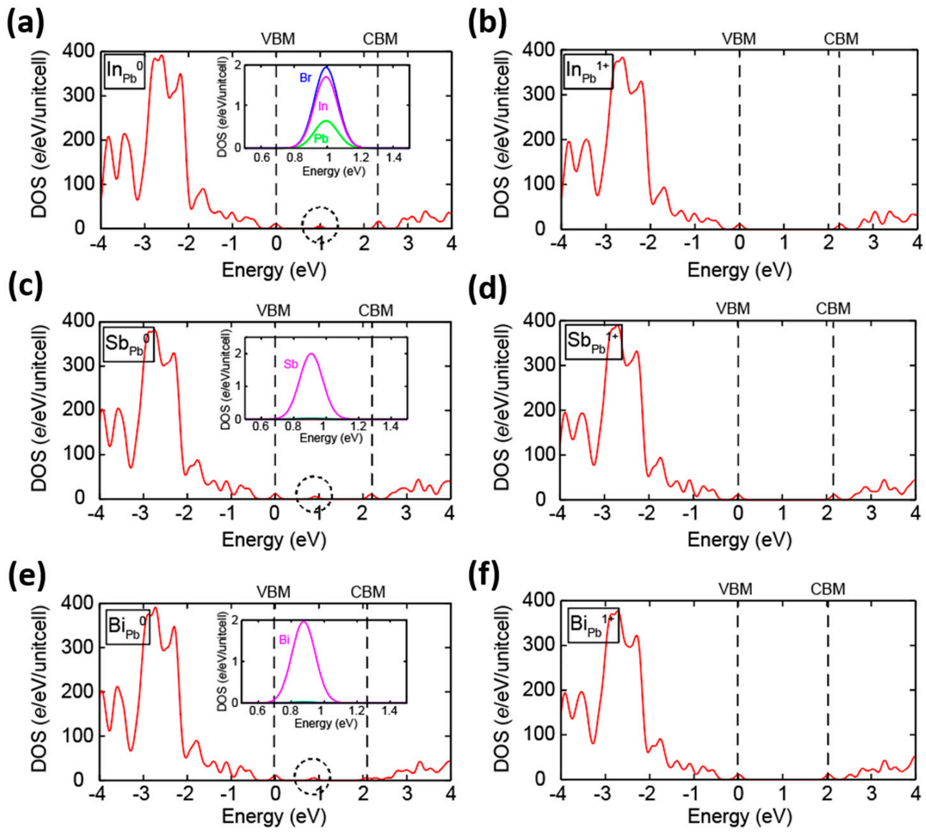

Figure S7. The electronic band structure and partial densities of states (DOS) of selected atoms such as (a) In_{Pb}^{0}, (b) In_{Pb}^{1+}, (c) Sb_{Pb}^{0}, (d) Sb_{Pb}^{1+}, (e) Bi_{Pb}^{0}, and (f) Bi_{Pb}^{1+}.

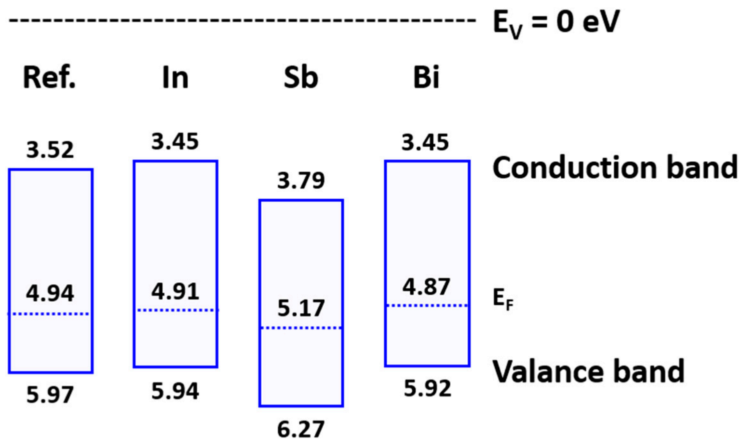

Figure S8. UPS spectra of pure and In-, Sb-, and Bi- ion substituted $CdPbBr_3$ perovskite QDs.

Table S1. gaps of binary bromide crystal calculated using PBE+SOC calculations methods.

	**InBr3**	**SbBr3**	**BiBr3**	**PbBr2**
Band gap (eV)	2.41	2.99	2.18	1.91

Table S2. composition ratio of atomic percentage of In-, Sb-, Bi- substituted $CsPbBr_3$ perovskite QDs.

[at%]

	**Cs**	**Pb**	**Br**	**In**	**Sb**	**Bi**
**Bi**	11.6	27.3	59.0	-	-	2.1
**Sb**	10.8	29.1	58.3	-	1.8	-
**In**	12.3	28.0	58.7	1.1	-	-
**Ref.**	13.5	27.9	58.5	-	-	-
